# Economic burden of dengue fever in China: A retrospective research study

**DOI:** 10.1371/journal.pntd.0010360

**Published:** 2022-05-20

**Authors:** Meng Xu, Nan Chang, Taotian Tu, Jimin Sun, Jinyong Jiang, Yu Xia, Wenge Tang, Hengqing Ji, Xiaotao Zhao, Jin Zhu, Li Qi, Xiaobo Liu, Qiyong Liu

**Affiliations:** 1 State Key Laboratory of Infectious Disease Prevention and Control, National Institute for Communicable Disease Control and Prevention, Chinese Center for Disease Control and Prevention, Beijing, China; 2 School of Public Health, Nanjing Medical University, Nanjing, Jiangsu, China; 3 Chongqing Center for Disease Control and Prevention, Chongqing, China; 4 Zhejiang Provincial Centre for Disease Control and Prevention, Hangzhou, Zhejiang, China; 5 Yunnan Institute of Parasitic Diseases, Pu’er, Yunnan, China; 6 Xishuangbanna Dai Autonomous Prefecture Center for Disease Control and Prevention, Xishuangbanna, Yunnan, China; 7 University College London, London, United Kingdom; Australian Red Cross Lifelood, AUSTRALIA

## Abstract

**Background:**

Dengue fever has been a significant public health challenge in China. This will be particularly important in the context of global warming, frequent international travels, and urbanization with increasing city size and population movement. In order to design relevant prevention and control strategies and allocate health resources reasonably, this study evaluated the economic burden of dengue fever in China in 2019.

**Methods:**

The economic burden of dengue fever patients was calculated from both family and the organisation perspectives. A survey was conducted among 1,027 dengue fever patients in Zhejiang, Chongqing, and Yunnan Provinces. Treatment expenses, lost working days, and insurance reimbursement expenses information were collected to estimate the total economic burden of dengue fever patients in 2019. The expenditures related to dengue fever prevention and control from government, Center for Disease Control and Prevention (CDC), communities and subdistrict offices of 30 counties (or districts) in Zhejiang Province and Chongqing City were also collected.

**Results:**

The direct, indirect and total economic burden for dengue fever patients in 2019 in the three Provinces were about 36,927,380.00 Chinese Yuan (CNY), 10,579,572.00 CNY and 46,805,064.00 CNY, respectively. The costs for prevention and control of dengue fever for the counties (or districts) without cases, counties (or districts) with imported cases, and counties (or districts) with local cases are 205,800.00 CNY, 731,180.00 CNY and 6,934,378.00 CNY, respectively. The total investment of dengue fever prevention and control in the 30 counties in China in 2019 was approximately 3,166,660,240.00 CNY.

**Conclusion:**

The economic burden of dengue fever patients is relatively high, and medical insurance coverage should be increased to lighten patients’ direct medical economic burden. At the same time, the results suggests that China should increase funding for primary health service institutions to prevent dengue fever transmission.

## Author summary

Dengue fever is one of the most important arbovirus diseases in the world. The distribution of cases has also increased significantly with the number of affected areas the highest in history. Dengue fever mainly occurs in southern provinces in China and its epidemic showed a trend of high incidence in 2019. This study estimated the economic burden of dengue fever in China in 2019 from the perspectives of both family and organisation. We recruited 1,027 dengue fever patients from Zhejiang Province, Chongqing City and Yunnan Province. At the same time, the costs related to dengue fever prevention and control from governments, CDCs, communities and subdistrict offices of 30 counties located in Zhejiang Province and Chongqing City were also collected. The results showed that in family perspective, the total economic burden of dengue in 2019 is estimated to be 46,805,064.00 CNY. In terms of organization expenditures, the regions with a higher incidence had higher costs.

## Introduction

Dengue fever is one of the most important arbovirus diseases in the world, prevalent in tropical and subtropical regions globally. In more than 100 countries and regions of Southeast Asia, the Pacific Islands and the Caribbean Sea, about half population is in the risk area [1–3]. In the past few decades, the spread of dengue fever has been intensified. The incidence of dengue fever has increased by 30 times in the past 50 years [4,5]. According to Global Burden of Disease (GBD), the global burden of dengue fever increased significantly from 8 million cases in 1990 to 50 million cases in 2013 [6]. According to the World Health Organization (WHO), dengue fever has become a serious global public health problem. About 2.5 billion people worldwide are at risk of dengue infection, with 3.2 million cases were reported each year in WHO Member States [7,8].

The first outbreak of dengue fever in China’s mainland occurred in Guangdong Province in 1978 [9]. Since then, dengue fever cases have been reported every year except for 1983, 1984, and 1996. In recent years, the spatial distribution of dengue fever cases and the risk of overseas import in China have gradually increased [10]. In 2019, China had a high incidence of dengue fever with more than 22,000 cases reported, second only to the 2014 outbreak. The geographic distribution of cases also increased significantly, and the number of affected areas was the highest in history [11]. Global warming, frequent international travels, urbanization with the extend of cities and population movement, the changes in mosquito breeding grounds and living habits, and the neglect of the management of the mosquito breeding environment all contributed dengue fever epidemic [11]. The challenge for dengue fever in China in the future may increase in the context of global warming which might have additional healthcare burden to already overloaded healthcare system. Therefore it is important to understand the current healthcare and prevention costs for dengue control and treatment so an effective disease control and health resource planning could be made to meet such challenges [12–14].

Previous studies had estimated the epidemiological burden of dengue fever in China and the economic burden of dengue fever patients in some areas [15–17], but the results were not comprehensive, with some studies only focused on the cost for patients without considering the those from dengue fever prevention and control [18]. Therefore, this study analyzed the economic burden of dengue fever from the perspectives of both organizations and families through field investigations, to provide comprehensive scientific evidence for decision-makers in their dengue fever prevention and control measures design and health resources allocation.

## Materials and methods

### Ethics statement

The study has been approved by the Ethics Committee of National Institute for Communicable Disease Control and Prevention, China. The approval number is ICDC-2019008. And the formal verbal consent has been obtained from the participants or the guardians of child participants.

A retrospective questionnaire survey was conducted to investigate the economic burden of dengue fever from both family and organization components. And all the participants signed the consent forms. Family economic burden refers to the economic loss borne by patients and their families, including direct medical expenses (diagnosis, treatment, medicines, consumables, consultation, hospitalization, self-purchased medicines, etc.), direct non-medical expenses (transportation, accommodation, caring and food costs for those away from hometowns) and indirect economic burden outside the scope of medical insurance (lost incomes from their family members due to absence of work). Organizational economic burden includes the costs of the treatment of dengue patients by government’s health insurance scheme and dengue preventative costs from other government organizations such as the Office of the National Patriotic Health Campaign Committee, Department of Health, the CDC, and local community. Specifically, government departments: costs of relevant meetings, printing promotional materials such as banners, slogans and advertisements, organizing and participating in training programs, coordinating input from other departments, hiring PEST Control Operation (PCO) companies. CDCs: labor fee for household investigation, printing publicity materials, hiring PCO companies, detection reagent and laboratory testing, training programs, sterilizing instrument and drug expenses, anti-mosquito equipment expenses, monitoring materials expenses, the cost for routine surveillance and emergency monitoring of density of *Aedes*, cost of case field investigation, vehicle fees, sterilizing fee of core area and warning area and other expenses. Local communities: costs of mosquito insecticides and instruments, costs of mosquito control equipment, sterilizing cost, labor fee for household investigation, Staff service fee, hiring PCO companies and other expenses.

### Study location

A random layer sampling method was used in this survey. All provinces, autonomous regions and municipalities in China were divided into high, medium, and low level according to the per capita Gross National Product (GDP) in 2018. Zhejiang Province, Chongqing City, and Yunnan Province were then selected as the survey sites accordingly. A multi-stage sampling approach was also adopted for the organizational component. The selection of provinces for the study of societal indirect burden was based on the epidemic situation of dengue fever in China in 2019, with one county (district) randomly selected from the one with traditional dengue outbreak and another from newly outbreak. The counties (districts) from the selected province or municipality were also divided into three levels for the survey: without cases, with only imported cases, and with local cases. In total, 30 counties (districts) totally from Zhejiang Province and Chongqing City were chosen as the survey sites.

### Survey approach

Relevant medical treatment costs for dengue fever patients were collected, after appropriate approvals, from hospital medical records or the medical insurance office. A questionnaire survey was undertaken to collect their non-medical expenses. Organisational operational costs were collected via electronic questionnaires survey.

### Data analysis

For patients’ burden, we calculated the direct economic burden by adding the direct and indirect medical expenses. The study used human capital approach to measure the indirect economic burden by multiplying the number of days that patients and their family members’ lost working days and daily per capita disposable income. The per capita disposable income was downloaded from the 2019 China Statistical Yearbook which income was calculated on a household basis and the unemployed or unpaid workers were included in the calculation. Finally, the number of cases at different levels was multiplied by the per capita economic burden of cases at different economic levels to calculate the family economic burden of dengue fever. Mann Whitney U test(two independent samples), Kruskal-Walls H test(three or more independent samples), and multivariate logistic regression were used to analyze the factors contributing to the family economic burden. In the multiple regression analysis, the data of economic burden of disease were normalized and then used as the dependent variable to construct the multiple linear regression equation.

For the organizational economic burden, we calculated it by adding the median costs of counties(districts) and the median cost of government’s health insurance scheme, and then multipling the number of dengue outbreaks counties(districts) at the corresponding levels at different economic level respectively to estimate the economic burden and value the total cost of the whole country. SPSS 22.0 was used for statistical analysis, with significant level at 0.05.

## Results

### Family economic burden of different types of patients

The survey sites were in Hangzhou City and Wenzhou City of Zhejiang Province, Jinghong City and Ruili City of Yunnan Province and Chongqing City. A total of 1,027 subjects were included, including 340 cases in Zhejiang Province, 350 cases in Chongqing City, and 337 cases in Yunnan Province. Among the 1,027 patients, 172 were outpatients, 855 were inpatients. There were 523 males and 504 females, with the average age 41.5 years.

After the Mann Whitney U test and Kruskal-Walls H test, the differences of medians had statistical significance in the economic burden of dengue fever patients with economic levels, ages, places of residence, family incomes, type of medical insurance, whether they have underlying diseases, and lost working days. For the indirect economic burden, the medians of the economic burden of dengue fever patients with different economic levels, ages, residences, types of medical insurance, and lost working days had significantly statistical differences (P<0.05) (Table 1). The results showed that the total economic burden of dengue fever patients who live in the urban and rural were 2549.00 CNY and 2139.00 CNY, respectively.

**Table 1:** Economic burden of patients with different types of dengue fever (CNY).

Item	Classification	Count	Direct economic burden			Indirect economic burden			Total economic burden		
			Median	Statistic	P-value	Median	Statistic	P-value	Median	Statistic	P-value
**Economic level**	high	340	2774.50	110.717	<0.001	882.00	130.228	<0.001	3690.50	161.117	<0.001
	medium	350	1541.00			432.00			1881.00		
	low	337	1413.00			385.00			1857.00		
**Gender**	male	523	1809.00	-1.742	0.081	440.00	-1.129	0.259	2328.00	-1.818	0.069
	female	504	1635.00			432.00			2110.00		
**Age**	0~	53	708.00	106.895	0.047	144.00	146.807	<0.001	916.00	116.076	0.012
	10~	55	1731.00			440.00			2171.00		
	20~	159	1685.00			412.50			2208.00		
	30~	218	1717.00			385.00			2047.00		
	40~	211	1421.00			385.00			1565.00		
	50~	172	1243.00			381.00			1706.00		
	60~	104	1250.00			385.00			1662.00		
	70~	42	1063.00			357.50			1492.00		
	≥80	13	1022.00			357.50			1357.00		
**Place of residence**	Urban	851	1673.00	-3.779	<0.001	432.00	-2.125	0.034	2139.00	-3.587	<0.001
	Rural	176	2036.00			495.00			2549.00		
**Household income**	0~	53	2241.00	17.510	0.002	385.00	4.938	0.294	2873.50	13.715	0.008
	15000~	180	1963.00			467.50			2498.00		
	50000~	324	1815.00			440.00			2417.50		
	100000~	227	1617.00			440.00			2181.00		
	≥150000	243	1480.00			440.00			1967.00		
**Type of medical insurance**	Public medical	10	2014.00	120.544	<0.001	819.00	18.171	0.006	2392.50	100.912	<0.001
	New rural cooperative medical	242	2527.50			504.00			3106.00		
	Basic medical insurance for urban residents	337	1300.00			385.00			1746.00		
	Urban employee insurance	264	1545.00			467.50			2080.50		
	Commercial insurance	8	2498.00			945.00			3898.00		
	Other	6	1951.00			882.00			2732.00		
	None	160	2247.00			440.00			2915.00		
**Basic illness**	Have	138	2185.50	-3.711	<0.001	504.00	-0.264	0.792	2668.50	-3.081	0.002
	None	889	1688.00			440.00			2148.00		
**Lost days**	0~	370	617.00	275.579	<0.001	72.00	867.268	<0.001	689.00	420.025	<0.001
	3~	197	658.00			144.00			834.50		
	6~	299	995.00			216.00			1211.00		
	9~	94	1161.50			288.00			1490.00		
	≥12	67	1631.50			360.00			2042.50		

### Multivariate analysis of family economic burden of dengue fever patients

In this study, the median values of direct, indirect, and total costs were used as the boundary values, those above the median were high (assigned 1), and those below the median (including the median) were low (assigned 0). Statistically significant variables in the univariate analysis were included as independent variables in the multivariate unconditional logistic regression model for analysis. The results showed that the factors of direct economic burden and total economic burden were economic level, age, medical insurance type and lost working days (P < 0.05). None of the variables had the statistically significant association with the indirect economic burden (Table 2).

**Table 2:** Multivariate logistic regression analysis of dengue fever patients’ economic burden.

	Direct economic burden				Indirect economic burden				Total economic burden			
	P-value	Odds Ratio	95% CI		P-value	Odds Ratio	95% CI		P-value	Odds Ratio	95% CI	
			Lower limit	Upper limit			Lower limit	Upper limit			Lower limit	Upper limit
**Economic level(control group:High)**	0				1				0			
**Medium**	0.101	0.713	0.475	1.069	0.982	0	0		0	0.343	0.217	0.542
**Low**	0	0.162	0.102	0.258	0.978	0	0		0	0.056	0.032	0.098
**Gender(control group:0~)**	0.045				0.462				0.029			
**10~**	0.231	1.803	0.687	4.737	0.991	1555268.597	0		0.559	1.373	0.475	3.972
**20~**	0.096	1.98	0.886	4.427	0.991	6596912.572	0		0.082	2.153	0.907	5.111
**30~**	0.368	1.441	0.651	3.189	0.992	1443485.85	0		0.831	1.097	0.467	2.579
**40~**	0.043	2.288	1.025	5.109	0.991	4747688.271	0		0.077	2.176	0.919	5.152
**50~**	0.077	2.126	0.922	4.904	0.992	1290503.505	0		0.227	1.756	0.705	4.376
**60~**	0.02	2.837	1.175	6.849	0.991	2733404.534	0		0.137	2.06	0.795	5.335
**70~**	0.002	5.653	1.908	16.753	0.985	1.342E+13	0		0.018	4.069	1.266	13.078
**≥80**	0.062	4.343	0.927	20.341	1	10220000000	0		0.018	7.197	1.411	36.707
**Place of residence(control group:Urban: Rural)**												
	0.29	1.297	0.801	2.101	0.464	0.482	0.069	3.389	0.125	1.534	0.888	2.651
**Household income(control group:0~)**	0.317				—	—	—	—	0.412			
**15000~**	0.153	0.556	0.249	1.242	—	—	—	—	0.124	0.501	0.208	1.209
**50000~**	0.351	0.692	0.319	1.5	—	—	—	—	0.295	0.637	0.274	1.481
**100000~**	0.242	0.627	0.287	1.371	—	—	—	—	0.232	0.593	0.252	1.396
**≥150000**	0.851	0.927	0.423	2.034	—	—	—	—	0.681	0.836	0.355	1.968
**Type of medical insurance(control group:Public medical)**	0				0.982				0			
**New rural cooperative medical**	0.255	2.332	0.543	10.01	0.994	0	0		0.472	1.844	0.347	9.784
**Basic medical insurance for urban residents**	0.636	0.702	0.162	3.042	0.994	0	0		0.477	0.543	0.101	2.923
**Urban employee insurance**	0.801	0.829	0.194	3.548	0.994	0	0		0.678	0.702	0.132	3.721
**Commercial insurance**	0.294	3.512	0.337	36.617	1	53.344	0		0.691	1.718	0.119	24.814
**Other**	0.218	5.323	0.373	76.066	1	61.874	0		0.45	3.059	0.168	55.715
**None**	0.007	7.849	1.75	35.206	0.993	0	0		0.052	5.458	0.988	30.138
**Basic illness(control group:Have)**					—	—	—	—				
**None**	0.293	0.773	0.479	1.249	—	—	—	—	0.084	0.62	0.36	1.067
**Lost days(control group:0~)**	0				1				0			
**3~**	0.231	1.352	0.826	2.212	0.986	9.11E+19	0		0.078	1.643	0.946	2.856
**6~**	0	4.709	2.918	7.6	0.981	4.278E+33	0		0	10.101	5.834	17.49
**9~**	0	6.547	3.693	11.608	0.977	6.301E+60	0		0	18.368	9.485	35.571
**≥12**	0	16.566	9.458	29.014	0.975	3.844E+60	0		0	118.179	55.526	251.524
**Constant**	0.243	0.336			0.985	0			0.628	0.599		

### Family economic costs

The medians of direct economic burden of each dengue fever patient at different economic levels from high to low were 2,774.50 CNY, 1,541.00 CNY and 1,413.00 CYN, and the indirect economic burden were 882.00 CNY, 432.00 CNY and 385.00 CNY, respectively. The total economic burden was 3,690.50 CNY, 1,881.00 CNY and 1,857.00 CNY. It was estimated that at the national level the direct economic burden of dengue fever in 2019 was 36,927,380.00 CNY, the indirect economic burden was 10,579,572.00 CNY, and the total economic burden was 46,805,064.00 CNY (Table 3).

**Table 3:** Family economic burden of dengue fever in China in 2019 (CNY).

Economic levels	Cases	Direct economic burden		Indirect economic burden		Total economic burden	
		Median	Total	Median	Total	Median	Total
**High**	2874	2774.50	7973913.00	882.00	2534868.00	3690.50	10606497.00
**Medium**	12847	1541.00	19797227.00	432.00	5549904.00	1881.00	24165207.00
**Low**	6480	1413.00	9156240.00	385.00	2494800.00	1857.00	12033360.00
**Total**	22201	36927380.00		10579572.00		46805064.00	

### Comparison of the direct economic burden of dengue fever patients from the organizational perspective and the family perspective

The study found that the medicare reimbursement (New rural cooperative medical, Basic medical insurance for urban residents, Urban employee insurance, Commercial insurance, and others) accounted for a large proportion of the total organizational investment of dengue fever. The mean of medicare reimbursement for patients in the high economic level was 1,271.00 CYN, for patients in the medium economic level was 1,013.00 CYN, and for patients in the low economic level was 997.00 CYN. It was estimated that the organizational cost of medicare reimbursement for dengue fever patients in 2019 was 23,127,425.00 CYN. Compared with the average cost and total cost for treatment from family perspective, the medicare reimbursement were lower. (Table 4).

**Table 4:** Direct medical expenses of dengue fever cases from different perspectives (CNY).

Economic level	Cases	Organizational perspective		Patient family perspective	
		Median	Total	Median	Total
**High**	2874	1271.00	3652854.00	2774.50	7973913.00
**Medium**	12847	1013.00	13014011.00	1541.00	19797227.00
**Low**	6480	997.00	6460560.00	1413.00	9156240.00
**Total**	22201	23127425.00		36927380.00	

### Expenses invested in the prevention and treatment of dengue fever

In this study, counties (districts) with no cases were classified as level one, with imported cases as level two, with local cases as level three. Level one included 3 counties (districts) in Zhejiang Province and 2 counties(districts) in Chongqing City. Level two included 6 counties (districts) in Zhejiang Province and 2 counties (districts) in Chongqing City. Level three included 11 counties(districts) in Zhejiang Province and 6 counties(districts) in Chongqing City. The cost of dengue prevention and control at all levels and departments were showed in Table 5.

**Table 5:** Organizational cost for prevention and treatment of dengue fever (CNY).

Item	Level One			Level Two			Level Three		
	Median	First Quartile	Third Quartile	Median	First Quartile	Third Quartile	Median	First Quartile	Third Quartile
**Government**	20000.00	17500.00	188850.00	117500.00	30778.75	457500.00	1972600.00	355940.00	5179145.00
**CDC**	190800.00	76895.00	277525.00	201900.00	103898.80	250750.00	617110.00	218350.00	1214610.00
**Street office, community**	—	—	—	682230.00	114390.00	3655563.00	4266387.50	964653.80	18485344.00
**Total**	205800.00	96895.00	466375.00	731180.00	270842.50	4177788.00	6934378.00	1409608.00	18964637.00

There were 290 counties(districts) with local cases, 1,099 counties(districts) with imported cases, and 1,711 with vector *Aedes* distribution totally in 2019 in China. It was estimated that China’s investment in dengue fever prevention and control in 2019 was about 3,166,600,240.00 CNY.

## Discussion

The economic burden caused by dengue fever control and treatment comprises an important part of healthcare costs in China. The results showed that, compared with the economic burden of other infectious diseases in China, the costs from dengue fever is lower. Liao [19] investigated the economic burden of patients from 21 notified infectious diseases in an infectious disease hospital and found that the average direct economic burden of patients from 2006 to 2015 ranged from 9,892.13 CNY to 12,572.06 CNY, while Wang et al. [20] found that the direct economic burden of Japanese encephalitis patients was 6889.00 CNY in Gansu Province, and it was 7228.00 CNY for scrub typhus patients [21]. The direct economic burden of all these diseases was higher than that of dengue patients in this study, and the indirect economic burden was similar. This could be due to the shorter duration of clinical symptoms and fewer severe cases of dengue fever when comparing to other chronic infectious diseases such as HBV infections.

The economic burden of dengue fever tends to be consistent with the regional economic development, areas with higher economic development have higher economic burdens for the disease treatment and prevention, with the direct economic burdens account for a larger proportion of the total cost of dengue fever, approximately 78.9%. The possible reasons are hospitals in developed areas may charge higher fees, local residents have higher incomes and are more willing to invest in medical care. Chen et al. [22] found that, in Ningbo, Zhejiang Province in 2018, the economic burden of dengue fever was 3,718.16 CNY, including the direct cost of 2,253.75 CNY and the indirect economic burden of 1,701.36 CNY. Another study in Guangdong Province showed that the cost of inpatients with dengue fever was US$499.64 [17]. The slight difference of results maybe caused by the cities with different economic levels selected in this study, so the average total economic burden is lower in our study.

In addition to economic level, age, type of medical insurance and lost working days also had influence on the direct and total economic burden. The age groups 70~ and 80~ were more likely to incur economic burden than age group 0~, with the OR increased gradually with age. This could be because that the symptoms are more severe and the treatment costs are higher among the elderly due to their poor physical condition and co-morbidities. Compared with public medical care patients, the probability of economic burden for patients with new rural cooperative medical insurance, commercial insurance, uninsured patients is higher, and for basic medical insurance for urban residents and urban employee insurance is lower. The dengue fever patients who live in rural areas and without basic health care have to pay higher medical costs.

Comparison of the direct economic burden of dengue fever between organizational and family perspectives showed that the direct family medical expenses were relatively high. Compared with the medicare reimbursement, most of the treatment costs were paid by the patient’s family. In Thailand, Vietnam, Colombia and other countries, direct medical expenses were relatively low for dengue treatment [23]. Especially in Thailand, the patient’s cost for of the burden of direct medical expenses is the smallest, only accounting for 2%. A study in Taiwan of China showed that the family direct burden of dengue fever was lower than indirect burden [16]. It is suggested that increase the reimbursement ratio of dengue fever-related treatment expenses should be considered to reduce the economic burden of patients.

From the organizational perspective, the economic burden of dengue fever in China in 2019 was about 3.2 billion CNY, accounting for 0.04‰ of China’s GDP. This result can provide a certain reference for the country’s funding and allocation for dengue prevention and control work. According to the results of the study, the investment of different departments in different levels of regions had increased successively, and regions with stronger epidemic intensity consumed more expenses than regions with weaker epidemic intensity.

Limitations of this study should be acknowledged. The study could only collect case data from general hospitals so the information from small-scale medical institutions such as township clinics could be missing. Additionally, for the counties (districts) without medical records, it is difficult to collect the cost from district and community institutions. Thirdly, the indirect economic burden of dengue fever on families was calculated by using the human capital approach. The numbers of lost working days reported in the questionnaire that patients and their family members are from their memories which recall biases may exist and the daily per capita disposable income in the 2019 China Statistical Yearbook were used. Respondents were asked to subjectively remove rest days and answer the number of lost working days when they lost their income. However, this method has certain limitations in the calculation of patients with unpaid work.

Recommendations could be made from our research findings. Firstly, community health education for dengue control and prevention should be promoted, especially for most vulnerable populations such as the elderly and higher incidence regions. Secondly, basic medical insurance system with the increased the coverage and proportion of reimbursement will help to reduce the burden on patients’ families. Thirdly, more funding for primary health service institutions to ensure the smooth progress of primary-level epidemic prevention work. Fourthly, more work should be focused on the areas with a large number of cases to ensure that infected patients can receive timely treatment, shorten the course of illness, and reduce hospitalization and severe cases to prevent more health resource consumption.
